# Dogs as Sources and Sentinels of Parasites in Humans and Wildlife, Northern Canada

**DOI:** 10.3201/eid1401.071113

**Published:** 2008-01

**Authors:** Amanda L. Salb, Herman W. Barkema, Brett T. Elkin, R.C. Andrew Thompson, Douglas P. Whiteside, Sandra R. Black, J.P. Dubey, Susan J. Kutz

**Affiliations:** * Calgary Zoo Animal Health Centre, Calgary, Alberta, Canada; † University of Calgary, Calgary, Alberta, Canada; ‡ Government of the Northwest Territories Wildlife Division, Yellowknife, Northwest Territories, Canada; § Murdoch University, Murdoch, Western Australia, Australia; ¶ US Department of Agriculture, Beltsville, Maryland, USA

**Keywords:** Dogs, parasites, zoonosis, aboriginal, wildlife, veterinary, Giardia, Toxoplasma, Arctic, dispatch

## Abstract

A minimum of 11 genera of parasites, including 7 known or suspected to cause zoonoses, were detected in dogs in 2 northern Canadian communities. Dogs in remote settlements receive minimal veterinary care and may serve as sources and sentinels for parasites in persons and wildlife, and as parasite bridges between wildlife and humans.

Throughout their long history of domestication, dogs have been sources of zoonotic parasites and have served as a link for parasite exchange among livestock, wildlife, and humans (*1*). Globally, dogs remain an important source of emerging disease in humans (e.g., eosinophilic enteritis caused by *Ancylostoma caninum*), a bridge for reemerging infections (*Echinococcus multilocularis*), and a source of parasites for immunocompromised persons (*1*).

Human disease and parasite infections in dogs in northern Canada have been recognized for some time (*2*–*5*). Historically, attention was focused on rabies virus, parvovirus, and canine distemper virus. However, dogs were also recognized as sources of zoonotic parasites such as *Echinococcus* spp. and as a possible bridge for rabies between wildlife and humans (*4*,*5*). Today, in many northern communities, veterinary services are absent or restricted, and disease surveillance programs and routine preventive health measures such as vaccination and parasite control are rare. These conditions have limited our understanding of disease interactions at the dog-human-wildlife interface and our ability to detect and respond to emerging diseases.

Northern environments and socioeconomic systems are changing rapidly and altering interactions among humans, animals, and their pathogens (*6*,*7*). In this study, we examined parasite diversity among dogs in 2 northern Canadian communities and evaluated the role of dogs as sentinels and sources of zoonotic infections in this changing landscape.

## The Study

Canine preventative healthcare clinics were available in Fort Chipewyan, Alberta, and Fort Resolution, Northwest Territories, in August 2006. Dogs were presented by their owners voluntarily and a detailed history, blood, and fresh fecal samples were obtained. Feces were stored at 4°C until examined within 6–12 days by quantitative sugar flotation and light microscopy (*8*). Fecal samples positive for *Giardia* spp. were genotyped (*9*). Serum samples were tested for antibodies against *Toxoplasma gondii* and *Neospora caninum* by using modified direct agglutination and immunofluorescence assays, respectively, at the US Department of Agriculture (Beltsville, MD, USA). Dilutions >1:25 were considered positive. Associations between parasitism and host (sex, age, community) and husbandry factors (housing, food type, community) were examined for adult dogs by χ^2^ analysis and Fisher exact test by using analytical software (Statistix, Tallahassee, FL, USA).

**Figure 1:**
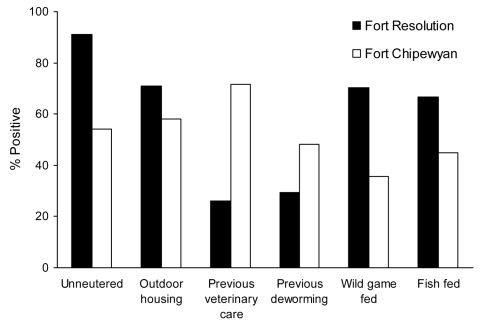
Husbandry practices for adult dogs (>6 months of age) in Fort Resolution and Fort Chipewyan, northern Canada. Results of all comparisons were significantly different between the 2 communities (p<0.05).

**Figure 2:**
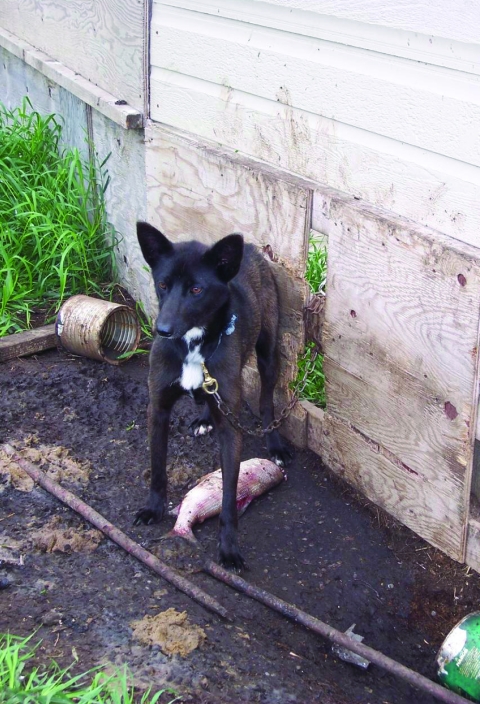
Northern dog with a typical meal. (Photograph provided by Susan J. Kutz.)

The study population consisted of a variety of breeds and cross-breeds, including Siberian husky, Laborador retriever, German shepherd, terriers, and other types. Most dogs were housed outdoors and many were fed fish and game (raw, frozen, fresh, cooked, or dry). Of dogs eating wild game, they ate moose (95.8%), muskrat (53.5%), caribou (54.9%), bison (45.1%), rabbit (28.2%), beaver (25.4%), elk (15.5%), and deer (14.1%) (Figures 1, 2).

A minimum of 11 parasite genera were detected and 47% of dogs had >1 gastrointestinal parasite (Tables 1, 2). Taeniid eggs were either from *Echinococcus* spp. or *Taenia* spp. Dogs housed outdoors were more likely to have housing-associated parasites such as *Toxocara* spp., *Toxascaris* spp., *Cystoisospora* spp., and *Uncinaria* spp. (p<0.0001). Dogs who ate wild game were more likely to have game-associated parasites such as *Sarcocystis* spp., *Taenia* spp., and *Echinococcus* spp. (p<0.05). No statistically significant associations were found between food or housing, and *T*. *gondii* or *N*. *caninum* infections and previous veterinary care or deworming had no effect on parasite prevalence.

**Table 1:** Prevalence and median intensity (range) of parasite eggs or oocysts in feces or positive titers for *Toxoplasma* and *Neospora* in dogs in 2 communities in northern Canada

Characteristic	Fort Chipewyan						Fort Resolution				
	Puppies			Adults			Puppies			Adults	
	M	F		M	F		M	F		M	F
No. dogs	6	1		32	20		5	7		37	21
Prevalence (%), median Intensity (range)*											
*Alaria* spp.†	0	0		0	0		0	0		14, 2 (1–134)	4, 7
*Diphyllobothrium* spp.†	17, 50	0		3, 2	0		20, 2	14, 1		11, 6 (1–603)	4, 6,429
Taeniid spp.†‡	0	0		6, 4 (2–5)	5, 55		0	0		11, 7 (1–770)	0
*Cytoisospora* spp.	0	0		0	5, 43		20, 30	0		0	4, 4
*Sarcocystis* spp.	0	0		0	15, 30 (3–53)		40, 2 (1–2)	14, 21		11, 13 (3–27)	13, 3 (1–255)
*Toxascaris* spp.†	0	0		0	5, 221		60, 251 (149–530)	29, 195 (93–297)		0	17, 138 (35–248)
*Toxocara* spp.†	33, 10,000 (610–20,000)	0		0	0		20, 6	14, 2		3, 11	9, 161 (1–321)
*Uncinaria* spp.†	0	0		47, 31 (1–333)	5, 63		20, 14	0		35, 40 (9– 251)	26, 27 (17–367)
No. dogs	4	1		30	13		3	7		23	15
*Giardia* spp.†	0	0		0	8		33	0		0	20
No. dogs	6	1		29	16		3	5		30	18
*Toxoplasma gondii* †§	50	100		41	50		100	80		60	56
*Neospora caninum*¶	0	0		3	6		0	0		7	0

*Range not reported if only 1 dog was positive. Intensity is the number of eggs or oocytes per gram of wet feces. †Zoonotic parasites. ‡*Echinococcus multilocularis, E. granulosus*, or *Taenia* spp. §23 dogs had titers of 25, 22 had titers of 50, 1 had a titer of 100, 1 had a titer of 200, and 1 had a titer of 400. ¶All 4 dogs had titers of 25.

**Table 2:** Percentage of dogs with multiple parasite genera detected by fecal flotation in 2 communities in northern Canada

Characteristic	Fort Chipewyan						Fort Resolution						Total				
	Puppies			Adults			Puppies			Adults			Puppies			Adults	
	M	F		M	F		M	F		M	F		M	F		M	F
Sample size	6	1		32	20		5	7		37	21		11	8		69	41
No. parasite genera																	
0	50	0		47	70		20	71		49	52		36	75		48	61
1	50	0		50	25		40	0		30	24		46	0		39	24
2	0	0		3	5		20	14		11	10		9	13		7	7
3	0	0		0	0		0	14		11	14		0	24		6	7
4	0	0		0	0		0	0		0	0		0	0		0	0
5	0	0		0	0		20	0		0	0		9	0		0	0
>2	0	0		3	5		40	29		22	24		18	25		13	15

## Conclusions

In the Northwest Territories, harvesting country foods is a key cultural activity and is important for sustenance; 75% of persons eat harvested meat and fish (*10*). Dogs fed fish and game can serve as indicators of parasites in these human food sources. Diet-associated zoonotic parasites detected in dogs included *Diphyllobothrium* spp., cestodes acquired by eating undercooked or inadequately frozen fish (found in humans throughout northern Canada); *Alaria* spp., trematodes acquired by eating frogs or paratenic hosts; and *T*. *gondii*, tissue protozoans acquired by eating oocysts from felid feces or tissue cysts in intermediate hosts (a worldwide human pathogen). In aboriginal persons in northern Canada, seroconversion for *T*. *gondii* during pregnancy has been associated with diets that include caribou (*11*). High seroprevalence in dogs indicates that *T*. *gondii* is common in the study area; however, the source of exposure was not identified. Given potential consequences for infection of parasite-negative pregnant women, further research is warranted on the association of human toxoplasmosis with a diet of country foods in northern regions.

*Toxocara* spp. are nematodes that cause visceral and ocular migrans in humans, particularly children. Although *Toxocara* spp. are considered limited to more southern regions (*3*), their presence in puppies and adults in Fort Resolution suggests that completion of their life cycle at northern latitudes is possible. Continuing warming trends may lead to increased occurrence of this parasite in the north. *Giardia* sp. Assemblage A is a protozoan that causes gastrointestinal disease in humans. Isolation of this zoonotic strain was unexpected because dogs are typically infected with Assemblage D, and Assemblage A suggests transmission from humans to dogs (*9*,*12*). This finding highlights a need to further investigate the apparent emergence of Assemblage A in domestic and wild animals in remote northern regions and transmission patterns among dogs, wildlife, and humans (S.J. Kutz, unpub. data). *Echinococcus* spp. are cestodes that cause hydatid (*E*. *granulosus*) or alveolar cysts (*E*. *multilocularis*) in the lungs and livers of humans. Although a reduction in dog teams in northern Canada has resulted in decreased prevalence of *E*. *granulosus* spp., the distribution, epidemiology, and role of the more pathogenic *E*. *multilocularis* spp. are not well understood in this region. *Uncinaria* spp. and *Toxascaris* spp. are also occasionally reported as zoonoses; however, evidence for these findings remains equivocal.

Dogs can also be sources of disease in parasite-naïve wildlife populations. They were the source for devastating distemper outbreaks in lions in the Serengeti (*13*), and lice of presumed dog origin are causing serious disease in Alaskan wolf populations (K.B. Beckmen, pers. comm.). *Neospora caninum* detected in this study may be a new parasite in this ecosystem with potentially serious consequences for wildlife. The remaining parasites are presumed present in local wildlife and can have a negative effect on the health of dogs and wildlife. More detailed, quantitative investigation is required to evaluate the role of dogs as potential sources of new, or amplifiers of existing, pathogens for wildlife.

Our results highlight important health issues associated with the interface between dogs, wildlife, and humans in remote northern communities. Disease associated with parasites in this study is often subclinical but can have serious effects on health and productivity of humans, dogs, and wildlife (e.g., *Giardia* spp.) (*14*). Although these parasites are relatively easy to control, there was no evidence that sporadic veterinary presence in Fort Chipewyan reduced parasitism. This finding emphasizes the need for a new approach to domestic animal healthcare in the north. Inaccessibility of communities, uncertain and changing roles of dogs, and current regulations in the veterinary profession restricting remote delivery of services hinder development of effective disease detection and preventative medicine programs. Innovative new methods for delivery of animal healthcare services are required. These methods should include long-term commitment to an integrated health approach, focusing on education, engagement, and development and support of local capacity for delivery of basic animal health services. Ongoing communication and partnerships between animal and human health professionals will enhance the effectiveness of such initiatives.
